# Resting-state functional magnetic resonance imaging shows altered brain network topology in Type 2 diabetic patients without cognitive impairment

**DOI:** 10.18632/oncotarget.21282

**Published:** 2017-09-27

**Authors:** Guan-Qun Chen, Xin Zhang, Yue Xing, Dong Wen, Guang-Bin Cui, Ying Han

**Affiliations:** ^1^ Department of Neurology, XuanWu Hospital, Capital Medical University, Beijing, China; ^2^ Center of Alzheimer’s Disease, Beijing Institute for Brain Disorders, Beijing, China; ^3^ National Clinical Research Center for Geriatric Disorders, Beijing, China; ^4^ Beijing Institute of Geriatrics, Beijing, China; ^5^ PKUCare Rehabilitation Hospital, Beijing, China; ^6^ Department of Radiology, Tangdu Hospital, The Fourth Military Medical University, Xi’an, China; ^7^ Radiological Sciences, Division of Clinical Neuroscience, Queen’s Medical Centre, University of Nottingham, Nottingham, United Kingdom; ^8^ Sir Peter Mansfield Imaging Centre, School of Medicine, University of Nottingham, Nottingham, United Kingdom; ^9^ School of Information Science and Engineering, Yanshan University, Qinhuangdao, China; ^10^ The Key Laboratory of Software Engineering of Hebei Province, Yanshan University, Qinhuangdao, China

**Keywords:** type 2 diabetes mellitus, cognitive dysfunction, functional connectivity, brain network, resting-state functional magnetic resonance imaging

## Abstract

We analyzed topology of brain functional networks in type 2 diabetes mellitus (T2DM) patients without mild cognitive impairment. We recruited T2DM patients without mild cognitive impairment (4 males and 8 females) and healthy control subjects (8 males and 16 females) to undergo cognitive testing and resting-state functional magnetic resonance imaging. Graph theoretical analysis of functional brain networks revealed abnormal small-world architecture in T2DM patients as compared to control subjects. The functional brain networks of T2DM patients showed increased path length, decreased global efficiency and disrupted long-distance connections. Moreover, reduced nodal characteristics were distributed in the frontal, parietal and temporal lobes, while increased nodal characteristics were distributed in the frontal, occipital lobes, and basal ganglia in the T2DM patients. The disrupted topological properties correlated with cognitive performance of T2DM patients. These findings demonstrate altered topological organization of functional brain networks in T2DM patients without mild cognitive impairment.

## INTRODUCTION

Diabetes mellitus (DM) is a global public health challenge that affected 415 million people worldwide in 2015 and 113.9 million Chinese in 2013 [1, 2]. Nearly 90% DM patients belong to type 2 diabetes mellitus (T2DM) category with a majority of these being elderly people. T2DM is a serious metabolic syndrome and a major risk factor for blindness, chronic kidney disease and ischemic stroke [2]. T2DM is also associated with mild cognitive impairment (MCI), which is a transitional state between normal aging and dementia [3–5]. However, the underlying mechanisms that accelerate cognitive decline or dementia are not clear.

The non-invasive, non-radioactive and operation-friendly resting-state functional magnetic resonance imaging (rs-fMRI) has offered valuable insights into understanding the neurophysiological mechanisms of cognitive disorders [6, 7]. It has been instrumental in identifying the role of T2DM in accelerating cognitive impairment [8–12]. T2DM patients with MCI show impaired functional connectivity in default mode network (DMN) [8, 9] and changed amplitude of low frequency fluctuations (ALFF) in the frontal lobe, temporal lobe, occipital lobe and amygdale [10–12]. These aberrant brain function patterns are closely associated with impaired cognitive performance [10, 11]. These methods analyze regional changes in brain function. However, cognitive function involves comprehensive interactions between different brain areas [13]. Hence, constructing the whole brain connectome is critical to understanding the underlying mechanisms of cognitive function and related disorders.

The human brain is a complex network characterized by a small-world network to achieve optimal cognitive function [14]. Graph theory-based network analysis is an effective method to investigate the topological organization of the human brain. It has been instrumental in understanding the underlying mechanisms of many brain diseases such as Alzheimer’s disease (AD), schizophrenia, multiple sclerosis, traumatic brain injury and epilepsy [14, 15]. For instance, the cognitive decline in AD patients is due to disrupted segregated and integrative connectivity patterns [16]. The graph theory-based network analysis also demonstrates altered topological organization of the brain network in T2DM patients with MCI [17–19]. However, it is not clear if altered topological organization of the brain network occurs prior to MCI in T2DM patients. Thus, it is necessary to detect the status of functional brain network organization in T2DM patients without cognitive impairment in order to identify the precise mechanisms in T2DM-related cognitive decline.

Therefore, in the present study, we analyzed if T2DM patients without cognitive impairment demonstrated disrupted organization of functional brain network. We also analyzed if changes in topological organization of the brain network correlated with cognitive performance. We hope it offers a new perspective on the understanding potential mechanism underlying the cognitive decline in T2DM patients.

## RESULTS

### Clinical and neuropsychological results

The age, sex, and education level of the T2DM patients and healthy controls were similar (*p* > 0.05; Table 1). Moreover, the MMSE, CDR and AVLT scores analyzing cognitive parameters were also similar for both groups (Table 1).

**Table 1:** Demographics and cognition scores of the study subjects

	Type 2 diabetes (*n =* 12)	Controls (*n =* 24)	*p* value
**Age** (years)	60∼74 (67.3 ± 4.7)	57∼76 (66.4 ± 5.5)	0.627
**Gender** (M/F)	4/8	8/16	1
**Education** (years)	1∼22 (12.0 ± 5.2)	0∼17 (11.3 ± 4.8)	0.670
**MMSE**	24∼30 (28.0±2.0)	20∼30 (27.8 ± 2.7)	0.816
**CDR**^a^ (0/0.5/1)	12/0/0	24/0/0	1
**AVLT**			
first immediate recall	4∼12 (6.8 ± 2.4)	4∼14 (6.9 ± 2.1)	0.873
second immediate recall	6∼14 (9.7 ± 2.4)	6∼14 (9.9 ± 2.1)	1
third immediate recall	7∼15 (10.8 ± 2.3)	7∼15 (11.3 ± 1.9)	0.489
average immediate recall	6.7∼13.3 (9.1 ± 1.8)	6.0∼13.7 (9.3 ± 1.7)	0.734
delayed recall	6∼14 (9.8 ± 2.5)	4∼15 (10.4 ± 2.6)	0.462
recognition	8∼15 (12.3 ± 2.2)	7∼15 (12.4 ± 2.3)	0.959

MMSE, mini-mental state examination; CDR, clinical dementia rating; AVLT, auditory verbal learning test. The values are expressed as range (mean ± sd). Note: ^a^ CDR = 0: Normal cognitive state; CDR = 0.5: Mild cognitive impairment (MCI); CDR=1: Dementia.

### Small-world properties of functional networks

**Figure 1:**
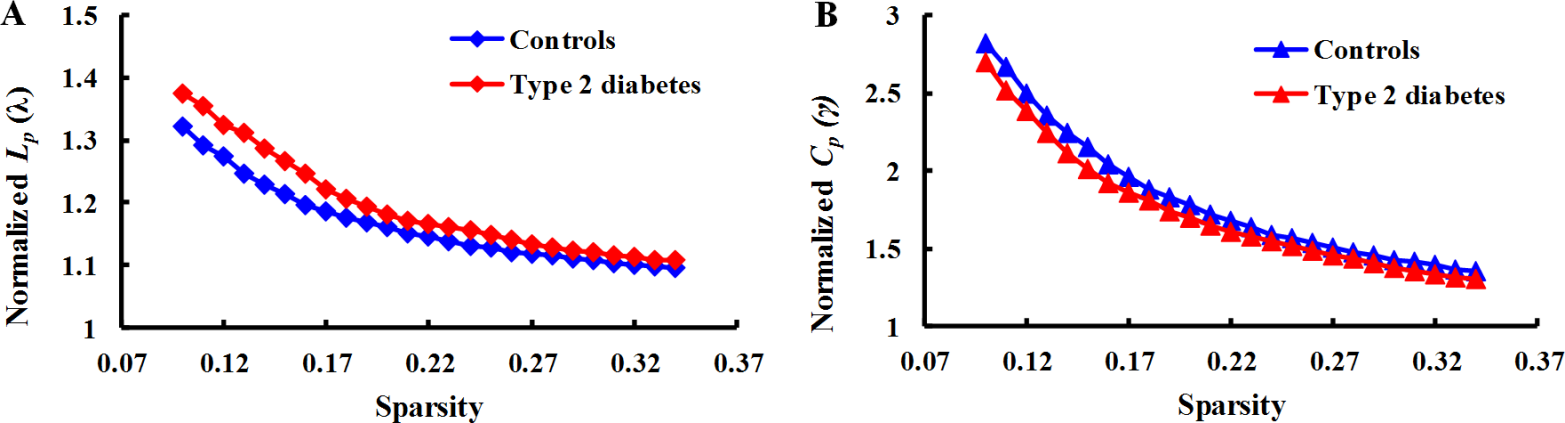
Network measures of functional brain networks for T2DM patients and normal controls (**A**) Graph plots showing mean normalized cluster coefficients (γ) for T2DM patients (red lines) and normal controls (blue lines) over a wide range of sparsity values (12–35%). (**B**) Graph plots showing mean normalized characteristic path length (λ) for T2DM patients (red lines) and normal controls (blue lines) over a wide range of sparsity values (12–35%). Networks of two groups have γ > 1 and λ ≈ 1, implying small-world properties.

Functional brain networks of the T2DM and control subjects had relatively high clustering coefficients (γ > 1) with identical characteristic path lengths (λ ≈ 1) compared to random networks, thereby demonstrating small-world property (Figure 1).

### Altered small-world property in T2DM patients without MCI

**Figure 2:**
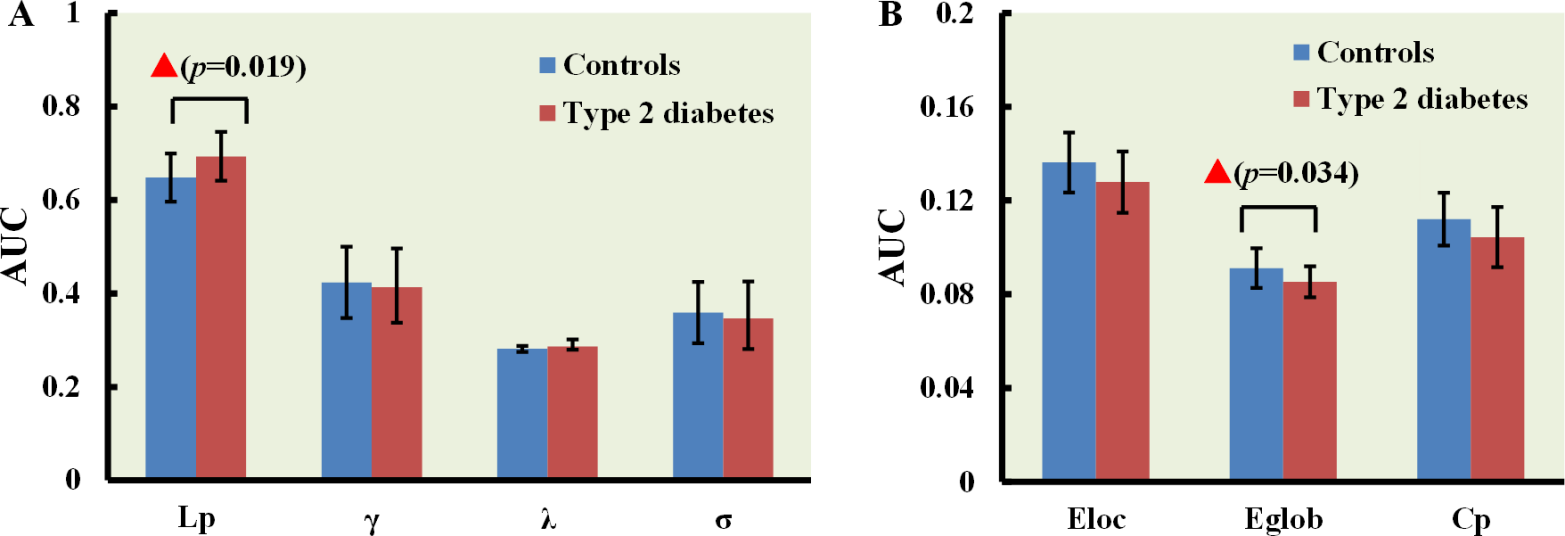
Small-world properties of T2DM patient and normal control brain networks (**A**) Bar graphs showing mean L_*p*_, γ, λ, and σ values for T2DM patients (red) and normal controls (blue). (**B**) Bar graphs showing mean E_*loc*_, E_*glob*_ and C_*P*_ values for T2DM patients (red) and normal controls (blue). The red triangle denotes *p* < 0.05 between the 2 groups. Error bars represent standard deviation from the mean.

We observed differences between T2DM and control subjects in some critical small-world properties and network efficiency parameters upon application of pre-defined threshold ranges. T2DM patients showed increased path length, L_p_ (*p* = 0.019) and decreased global efficiency, E_glob_ (*p* = 0.034) in comparison to control subjects (Figure 2). However, C_P_, E_loc_, γ, λ, and σ values were similar between T2DM patients and normal controls (Figure 2).

### Altered regional nodal characteristics in T2DM patients without MCI

The brain regions showing differences (*p* < 0.05) between T2DM and control subjects in at least one of the three nodal characteristics are summarized in Table 2. We identified 21 brain regions with altered nodal metrics. T2DM patients showed decreased nodal characteristics in frontal [bilateral superior frontal gyrus (orbital part), left middle frontal gyrus, left middle frontal gyrus (orbital part), left frontal gyrus (orbital part), bilateral olfactory cortex, right gyrus rectus], parietal (bilateral superior parietal gyrus, left paracentral lobule) and temporal (left parahippocampal gyrus, bilateral inferior temporal gyrus) lobes compared to normal controls. However, T2DM patients showed increased nodal characteristics in the left inferior frontal gyrus (triangular part), left gyrus rectus, left posterior cingulate gyrus, right calcarine fissure and surrounding cortex, right cuneus, right supramarginal gyrus, and right lenticular nucleus (putamen) compared to control subjects (Table 2).

**Table 2:** Regions with altered nodal characteristics in type 2 diabetes patients

AAL No.	Brain Regions	*p* values		
		Nodal Degree	Nodal Efficiency	Nodal Betweeness
**T2DM<NC (14/21)**				
5	Left superior frontal gyrus, orbital part	**0.005**	**0.004**	0.374
6	Right superior frontal gyrus, orbital part	**0.005**	**0.001**	0.512
7	Left middle frontal gyrus	0.073	**0.016**	0.503
9	Left middle frontal gyrus, orbital part	**0.007**	**< 0.001**	0.411
15	Left frontal gyrus, orbital part	**0.009**	**0.005**	**0.012**
21	Left olfactory cortex	**0.005**	**< 0.001**	0.132
22	Right olfactory cortex	**0.001**	**< 0.001**	0.530
28	Right gyrus rectus	0.066	**0.019**	0.510
39	Left parahippocampal gyrus	0.145	0.099	**0.014**
59	Left superior parietal gyrus	0.133	**0.030**	**0.030**
60	Right superior parietal gyrus	0.108	**0.033**	0.319
69	Left paracentral lobule	0.182	0.257	0.017
89	Left inferior temporal gyrus	0.071	**0.035**	0.446
90	Right inferior temporal gyrus	**0.017**	**0.012**	0.361
**T2DM>NC (7/21)**				
13	Left inferior frontal gyrus, triangular part	0.343	0.499	**0.009**
27	Left gyrus rectus	0.485	0.497	**0.037**
35	Left posterior cingulate gyrus	0.496	0.505	**0.027**
44	Right calcarine fissure and surrounding cortex	0.129	0.319	**0.040**
46	Right cuneus	0.243	0.384	**0.031**
64	Right supramarginal gyrus	0.118	0.478	**0.042**
74	Right lenticular nucleus, putamen	**0.043**	0.255	0.489

AAL No., number of automated anatomical labeling. Note: regions were considered abnormal in T2DM patients if they exhibited *p* < 0.05 compared to normal subjects in at least one of the three nodal characteristics (shown in bold).

### Regions with altered inter-regional functional connectivity in T2DM patients

**Figure 3:**
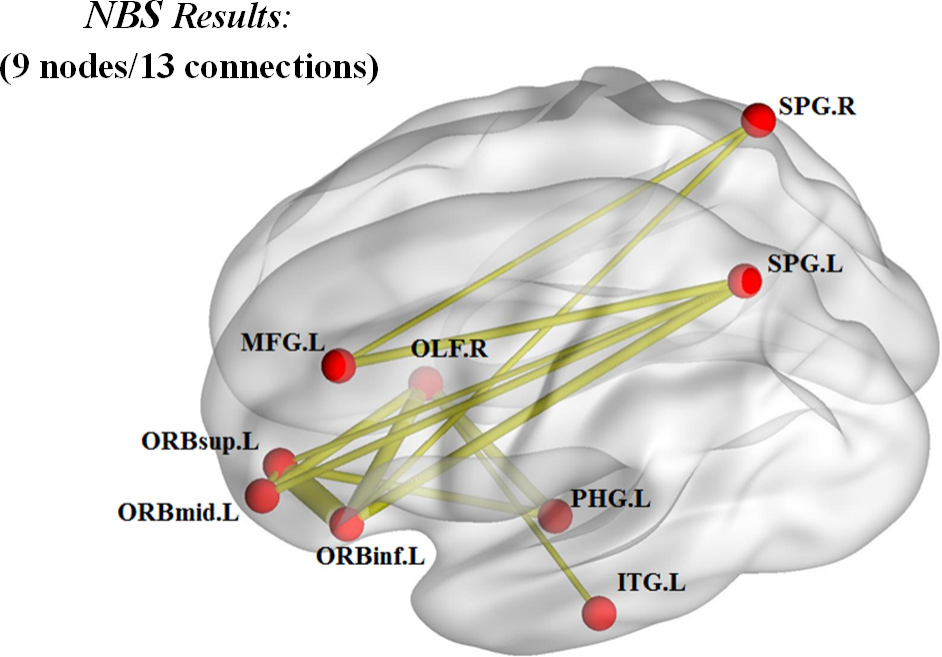
Brain network connections in T2DM patients with decreased values The brain regions with decreased functional connections in T2DM patients are shown. These connections form a connected network with 9 nodes and 13 connections. Seven of the 13 connections are long-distance connections (larger than 75 mm) that link different lobes.

NBS analysis identified 21 altered nodes between the T2DM and control subjects [20]. Among these, nine nodes had 13 altered connections in T2DM patients. These included the left superior frontal gyrus (orbital part), left middle frontal gyrus (orbital part), left inferior frontal gyrus (orbital part), left middle frontal gyrus, left parahippocampal gyrus, left inferior temporal gyrus, left superior parietal gyrus, right olfactory cortex, and right superior parietal gyrus (Table 3). Among these 13 connections, seven were long-distance connections (larger than 75 mm) that linked different lobes (Figure 3). Table 4 shows connections with decreased strength in T2DM patients compared to normal controls.

**Table 3:** Regions with altered inter-regional functional connectivity in T2DM patients identified by NBS analysis

AAL No.	Brain Regions
5	Left superior frontal gyrus, orbital part (ORBsup.L)
9	Left middle frontal gyrus, orbital part (ORBmid.L)
15	Left inferior frontal gyrus, orbital part (ORBinf.L)
7	Left middle frontal gyrus (MFG.L)
39	Left parahippocampal gyrus (PHG.L)
89	Left inferior temporal gyrus (ITG.L)
59	Left superior parietal gyrus (SPG.L)
22	Right olfactory cortex (OLF.R)
60	Right superior parietal gyrus (SPG. R)

**Table 4:** Altered inter-regional functional connections in type 2 diabetes patients compared with normal controls identified by NBS analysis

AAL No.	Region A	Region B	Mean FC difference (FC_diabetes_−FC_control_)	Euclidean distance (mm)
5–9	Left superior frontal gyrus, orbital part	Left superior frontal gyrus, orbital part	−0.173	14.888
5–15	Left superior frontal gyrus, orbital part	Left inferior frontal gyrus, orbital part	−0.245	25.591
5–39	Left superior frontal gyrus, orbital part	Left parahippocampal gyrus	−0.200	63.873
5–59	Left superior frontal gyrus, orbital part	Left superior parietal gyrus	−0.215	129.204
7–59	Left middle frontal gyrus	Left superior parietal gyrus	−0.183	95.757
7–60	Left middle frontal gyrus	Right superior parietal gyrus	−0.162	112.696
9–22	Left superior frontal gyrus, orbital part	Right olfactory cortex	−0.197	53.676
9–59	Left superior frontal gyrus, orbital part	Left superior parietal gyrus	−0.183	129.815
15–22	Left inferior frontal gyrus, orbital part	Right olfactory cortex	−0.225	48.721
15–59	Left inferior frontal gyrus, orbital part	Left superior parietal gyrus	−0.182	115.567
15–60	Left inferior frontal gyrus, orbital part	Right superior parietal gyrus	−0.187	132.047
22–39	Right olfactory cortex	Left parahippocampal gyrus	−0.238	45.858
22–89	Right olfactory cortex	Left inferior temporal gyrus	−0.180	75.488

FC, functional connectivity; AAL No., number of automated anatomical labeling. Note: Region A and Region B refers to the nodes corresponding to each altered connection.

### Correlation analysis between global network properties and behavioral measures

**Figure 4:**
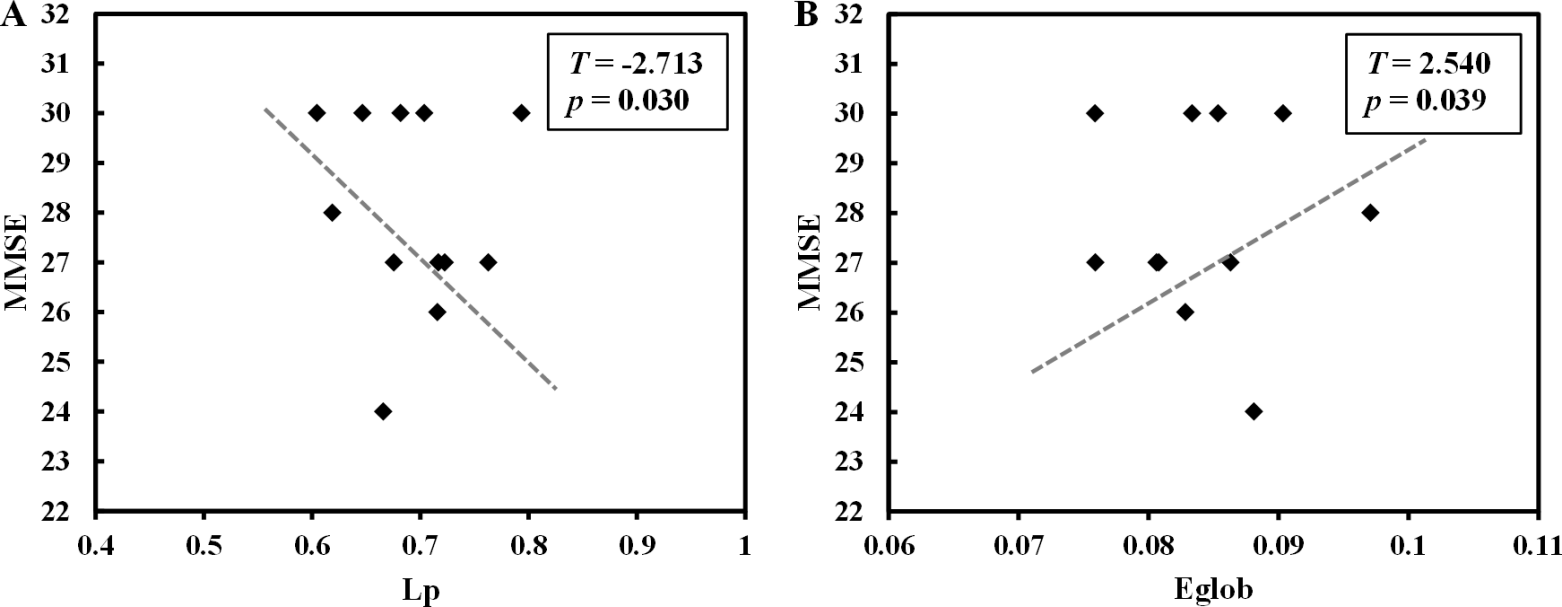
Correlation between global network properties and behavioral measures (**A**) Mean negative correlation between L_*p*_ and MMSE scores (*p* = 0.030, adjust R^2^ = 0.645) in T2DM patients. (**B**) Mean positive correlation between E_*glob*_ and MMSE scores (*p* = 0.039, adjust R^2^ = 0.621) in T2DM patients.

In T2DM patients, L_*p*_ showed a negative correlation with MMSE scores (*p* = 0.030, adjust R^2^ = 0.645), whereas E_*glob*_ showed a positive correlation (*p* = 0.039, adjust R^2^ = 0.621; Figure 4). In control subjects, there was no correlation between any global network properties and behavioral measures.

## DISCUSSION

In this study, the rs-fMRI data showed alterations in the global functional organization and connectivity in the brain network of T2DM patients without cognitive impairment. The T2DM patients without cognitive impairment showed (1) functional brain networks or small-world properties like normal controls; (2) longer characteristic path lengths and reduced global efficiency; (3) abnormal nodal characteristics and functional connectivity pairs; and (4) disrupted network topological properties related to MMSE. Together, these findings suggested altered functional brain networks in T2DM patients prior to MCI. Moreover, our study showed that graph theory-based network analysis could identify the nature of the cerebral changes underlying the diabetes-related cognitive decline.

A small-world network involves a high clustering coefficient (a measure of local network connectivity) and short characteristic path length (a measure of global network connectivity) in combination [21]. This combination provides an optimal type of architecture for the segregation and integration of information. Small-world networks have been found in several real-world networks including genetic and metabolic networks as well as social and economic systems [14]. Many studies have analyzed structural and functional human brain networks with non-invasive neuroimaging technologies such as MRI, EEG, and MEG [22–26]. These studies recognized that human brain networks were small-world networks. From an evolutionary perspective, brain network organization maximized efficiency and minimized information processing [27]. In our study, brain networks in both healthy controls and T2DM patients were characteristic of small-world networks (Figure 1).

Our investigation revealed that the topological properties of the brain network were altered in T2DM patients without cognitive impairment (Figure 2), consistent with previous studies [17–19]. The characteristic path length (L_p_) is a measure of global network connectivity and is defined as the minimum number of edges between any two nodes [27]. In brain networks, a relatively short characteristic path length ensures integration and transmission of information between and across brain regions and forms the basis for cognition [28]. Longer path lengths are associated with the impaired cognitive functions (as measured with MMSE), which indicate disrupted integration among the distant neurons [29]. Global efficiency (E_glob_) is inversely proportional to the average path length [27]. In this study, T2DM patients showed increased path length and decreased global efficiency in the functional brain networks before detecting MCI. Since small-world network is optimally organized for segregating and integrating information, the longer path length and decreased global efficiency in the T2DM networks indicates altered functional organization. A previous study on major depressive disorder patients showed that increased long distance functional connections lead to shorter path lengths [30]. This was analogous to decreased long distance functional connections in T2DM patients without cognitive impairment (Figure 3; Table 4). Our findings are partly in accordance with other studies that investigated changes of structural network (white matter) in T2DM patients with MCI [17, 18]. However, T2DM patients with MCI showed other disrupted topological organization of the white matter network including clustering coefficient, local efficiency, and network strength as well as increased path length and declined global efficiency. These differences demonstrated severity of the disease condition. We postulate that topological properties of functional brain network worsen as the pathology becomes more severe. This may be the reason for normal cluster coefficient and local efficiency in T2DM patients without cognitive impairment. However, further studies with longer follow-up times are necessary to confirm these findings.

Following the discovery of a disrupted global network organization in T2DM, we further localized brain regions exhibiting altered nodal characteristics (nodal degree, nodal efficiency, and nodal betweeness). T2DM patients showed regions with altered nodal characteristics in frontal, parietal and temporal lobes (Table 2). Most (14/21) regions showed decreased nodal characteristics in T2DM. A previous study showed impairment of hippocampus-mediated episodic memory in most T2DM patients as measured with AVLT-delayed recall and AVLT-recognition [8]. Furthermore, voxel-based morphometry analysis showed cortical atrophy in the temporal lobe of T2DM patients [31]. Our results showed decreased nodal characteristics in the temporal lobe [left parahippocampal gyrus, left inferior temporal gyrus and right inferior temporal gyrus], thereby suggesting gray matter loss. In addition to memory deficits, attention impairment was another common manifestation of T2DM related cognition impairment [32, 33]. Functional connectivity analysis revealed that dorsal attention network, which is an attention-related functional network was disrupted in T2DM patients [34]. In the present study, as shown in Table 2, several attention-related cortex regions [left superior frontal gyrus, orbital part, right superior frontal gyrus, orbital part, left middle frontal gyrus, left middle frontal gyrus, orbital part, left frontal gyrus, orbital part, left superior parietal gyrus, and right superior parietal gyrus] showed decreased nodal characteristics. Therefore, reduced nodal characteristics maybe attributed to disrupted attention network.

Olfactory cortex is associated with olfaction, and olfactory dysfunction has been reported in diabetes [35, 36]. Moreover, neuropathic pain can be partly explained by olfactory dysfunction in DM patients [37]. In the current study, bilateral olfactory cortex of T2DM patients showed decreased nodal characteristics, which may contribute to diabetes-related olfactory dysfunction. However, this needs to be further investigated since we did not conduct olfactory function tests in this study. In addition, T2DM patients showed increased functional connectivity in various brain regions [9, 10, 38, 39]. We hypothesize that increased nodal characteristics of the cortical regions in T2DM are compensation for the reduced characteristics in the other brain regions. Together, our results suggest alterations in brain regions and functional networks in T2DM patients without cognitive decline.

MMSE is a simple, widely used neuropsychological test for assessing general cognitive function [40]. Although both T2DM and control subjects had similar MMSE scores, specific disrupted network topological properties were relevant to MMSE in T2DM patients (Figure 4). In particular, L_*p*_ was negative correlated and E_*glob*_ was positively correlated with MMSE scores. This indicated that as T2DM progressed, increased L_*p*_ and decreased E_*glob*_ lead to cognitive dysfunction. Numerous studies have reported T2DM patients with declined cognition in multiple domains including processing speed, memory, executive functioning, and verbal fluency [32, 41]. However, further studies are necessary to analyze if disrupted network topological properties contribute to cognitive impairment of T2DM patients as shown in our study.

Our study has some limitations. First, the study had small sample size and did not assess progression of functional brain network changes. Second, T2DM diagnosis was self-reported based on history of diabetes and medication records. Therefore, effects of several factors including levels of plasma glucose, HbA1c and serum insulin on brain network properties as well as our findings are unknown. Moreover, most of the T2DM patients had received medication, so the results might be confounded by the medication effect. Therefore, the effect of medication is a subject that needs to be explored in future studies. Finally, functional brain networks depend on brain structural pathways. Therefore, structural MRI data is necessary to examine if functional network changes in T2DM are associated with structural network alterations.

In summary, our study showed that functional brain networks of T2DM patients without cognitive impairment had altered small-world properties suggesting that abnormal brain function preceded cognitive dysfunction in T2DM patients. Therefore, studies on cognitive impairment caused by AD should exclude participants with a history of T2DM when enrolling healthy controls. Furthermore, our study indicated that graph theory-based network analysis is a powerful tool to understand the underlying mechanisms of DM-related cognitive decline.

## MATERIALS AND METHODS

### Study subjects

This study was approved by the medical research ethics committee and institutional review board of XuanWu Hospital, Capital Medical University of China and adhered to the rules of the Declaration of Helsinki. Informed consent was obtained from all study subjects. We enrolled 36 right-handed subjects including 12 T2DM patients (4 males and 8 females) and 24 sex-, age-, and education-matched normal control subjects (8 males and 16 females) from the neurology department of Xuan Wu hospital (Beijing, China).

A standardized diagnostic evaluation was performed for all study subjects including a medical history interview, physical and neurological examinations, laboratory tests, brain MRI neurological examination and neuropsychological tests. Participants were categorized as diabetic if they reported a history of diabetes and took diabetes medication. Control individuals with fasting glucose > 6.1 mM or postprandial glucose > 7.8 mM were excluded from this study.

Exclusion criteria included history of (1) stroke; (2) psychiatric disease; (3) neurological disorder; (4) alcohol or drug abuse; and (5) systemic diseases like severe anemia, thyroid dysfunction, syphilis, or Acquired Immune Deficiency Syndrome.

### Neuropsychological test

Detailed standardized cognitive assessment was performed on all study participants and their scores were found to be within the normal range. The Chinese version of the Mini-Mental State Examination (MMSE) [42], clinical dementia rating (CDR) [43], and the auditory verbal learning test (AVLT) [44] were used to evaluate cognitive function. All subjects showed normal performance on MMSE after adjusting for education and the scores of CDR were 0.

### Image acquisition

Brain imaging was performed for all participants with a 3-T Trio Siemens scanner at XuanWu Hospital, Capital Medical University of China. Resting state functional MRI (rs-fMRI) images were collected using an echo-planar imaging (EPI) sequence with the following parameters: repetition time (TR) = 2000 ms; echo time (TE) = 40 ms; flip angle = 90°; number of slices = 28; slice thickness = 4 mm; gap = 1 mm; voxel size=4 × 4 × 4 mm^3^; and matrix = 64 × 64. Participants lay quietly with their eyes closed during the data acquisition in the scanner. Each scan lasted 478 seconds. In addition, 3D T1 scans and diffusion images were obtained for all participants, but were not used in the current study.

### Data preprocessing

Pre-processing and network construction of rs-fMRI images was performed with GRETNA package (http://www.nitrc.org/projects/gretna). The first 10 time-points of the fMRI series were excluded to avoid instability of the initial MRI signal. The images were normalized into a 3 × 3× 3 mm^3^ Montreal Neurological Institute (MNI) 152 template after slice timing and head motion correction and spatially smoothened with a 4 mm full-width half-maximum (FWHM) three-dimensional Gaussian kernel. Then, the images were linearly detrended and temporally band-pass filtered (0.01∼0.08 Hz). Finally, the global, the white matter (WM) and the cerebrospinal fluid (CSF) signals as well as the motion parameters were regressed out.

We constructed the whole-brain network with the automated anatomical labeling (AAL) atlas. First, the whole brain was divided into 90 different brain regions and the functional connectivity between each pair of regions calculated. Each region represented one node of the brain network. The edges were determined with a pre-defined threshold on the functional connectivity strength. Specifically, if the connectivity strength was greater than the threshold between the corresponding nodes, it was counted as one link or node. Thus, the brain network was an undirected graph with 90 nodes and corresponding edges. To address the issue of single threshold selection, few sparsity thresholds ranging from 0.1∼0.34 with an interval of 0.01 were applied and the remaining fraction of edges were calculated in the network for each study subject [30, 45]. For each sparsity threshold, ten global and node network metrics were computed. The global metrics included five small-world parameters (clustering coefficient C_p_, characteristic path length L_p_, normalized clustering coefficient γ, normalized characteristic path length λ, and small-worldness σ) and two network efficiency parameters (local efficiency E_loc_ and global efficiency E_glob_). The node metrics included nodal degree, nodal efficiency and nodal betweenness. The area under curves (AUC), which are independent of single threshold selection and sensitive to topological alterations in other brain disorder studies were analyzed for each parameter over the entire sparsity range [30, 45].

### Statistical analysis

Differences between study groups was determined by one-tailed non-parametric permutation tests performed on both global and nodal networks. AUC metrics were determined using the Matlab platform. Briefly, mean value of each network metric was calculated for both groups. Then, values were randomly allocated for each metric in the two groups and the mean differences were recomputed. This randomized reallocation procedure was repeated 10,000 times and the 95th percentile of each distribution was used as the critical value for the one-tailed test with a null hypothesis with a type I error of 0.05.

To locate the specific pairs of region showing altered functional connectivity, we performed the network-based connections (NBS) method (http://www.nitrc.org/projects/nbs/) [20]. This method detected pairs with significant differences between the two groups in at least one of the three nodal metrics (node degree, efficiency, and betweeness). In brief, we generated a subset of connection matrix composed of the above pair connections and NBS method was used to identify a set of suprathreshold links among any of the connected components (threshold, T = 2.441, *P* < 0.01). Again, non-parametric permutation tests were used to determine if the functional connectivity between those pairs was significantly altered (10,000 permutations, *P* < 0.05).

Moreover, we used multiple linear regression analyses with age, gender and education as confounding factors to assess the relationships between the abnormal global network metrics and the MMSE and AVLT scores, respectively.

## Abbreviations

AAL: automated anatomical labeling
AD: Alzheimer’s disease
AVLT: auditory verbal learning test
ALFF: amplitude of low frequency fluctuations
AUC: area under curve
CSF: cerebrospinal fluid
CDR: clinical dementia rating
DMN: default mode network
EPI: echo-planar imaging
FC: functional connectivity
FWHM: full-width half-maximum
rs-fMRI: resting-state functional magnetic resonance imaging
MCI: mild cognitive impairment
MMSE: Mini-Mental State Examination
MNI: Montreal Neurological Institute
NBS: network-based connections
T2DM: type 2 diabetes mellitus
TR: repetition time
TE: echo time
WM: white matter

## References

[1] IDF Diabetes Atlas - Seventh Edition (2015).

[2] Xu Y, Wang L, He J, Bi Y, Li M, Wang T, Wang L, Jiang Y, Dai M, Lu J, Xu M, Li Y, Hu N (2013). Prevalence and control of diabetes in Chinese adults. Jama.

[3] Biessels GJ, Staekenborg S, Brunner E, Brayne C, Scheltens P (2006). Risk of dementia in diabetes mellitus: a systematic review. Lancet Neurol.

[4] Cukierman T, Gerstein HC, Williamson JD (2005). Cognitive decline and dementia in diabetes—systematic overview of prospective observational studies. Diabetologia.

[5] Luchsinger JA, Reitz C, Patel B, Tang MX, Manly JJ, Mayeux R (2007). Relation of diabetes to mild cognitive impairment. Arch Neurol.

[6] Liu Z, Zhang Y, Yan H, Bai L, Dai R, Wei W, Zhong C, Xue T, Wang H, Feng Y, You Y, Zhang X, Tian J (2012). Altered topological patterns of brain networks in mild cognitive impairment and Alzheimer’s disease: A resting-state fMRI study. Psychiatry Research: Neuroimaging.

[7] Liu Z, Zhang Y, Bai L, Yan H, Dai R, Zhong C, Wang H, Wei W, Xue T, Feng Y, You Y, Tian J (2012). Investigation of the effective connectivity of resting state networks in Alzheimer’s disease: a functional MRI study combining independent components analysis and multivariate Granger causality analysis. Nmr Biomed.

[8] Zhou H, Lu W, Shi Y, Bai F, Chang J, Yuan Y, Teng G, Zhang Z (2010). Impairments in cognition and resting-state connectivity of the hippocampus in elderly subjects with type 2 diabetes. Neurosci Lett.

[9] Chen YC, Jiao Y, Cui Y, Shang SA, Ding J, Feng Y, Song W, Ju SH, Teng GJ (2014). Aberrant brain functional connectivity related to insulin resistance in type 2 diabetes: a resting-state fMRI study. Diabetes Care.

[10] Xia W, Wang S, Sun Z, Bai F, Zhou Y, Yang Y, Wang P, Huang Y, Yuan Y (2013). Altered baseline brain activity in type 2 diabetes: a resting-state fMRI study. Psychoneuroendocrino.

[11] Zhou X, Zhang J, Chen Y, Ma T, Wang Y, Wang J, Zhang Z (2014). Aggravated cognitive and brain functional impairment in mild cognitive impairment patients with type 2 diabetes: a resting-state functional MRI study. J Alzheimers Dis.

[12] Cui Y, Jiao Y, Chen YC, Wang K, Gao B, Wen S, Ju S, Teng GJ (2014). Altered spontaneous brain activity in type 2 diabetes: a resting-state functional MRI study. Diabetes.

[13] Delbeuck X, Van der Linden M, Collette F (2003). Alzheimer’s disease as a disconnection syndrome?. Neuropsychol Rev.

[14] Stam CJ (2014). Modern network science of neurological disorders. Nat Rev Neurosci.

[15] He Y, Evans A (2010). Graph theoretical modeling of brain connectivity. Curr Opin Neurol.

[16] He Y, Chen Z, Gong G, Evans A (2009). Neuronal networks in Alzheimer’s disease. Neuroscientist.

[17] Reijmer YD, Leemans A, Brundel M, Kappelle LJ, Biessels GJ (2013). Disruption of the cerebral white matter network is related to slowing of information processing speed in patients with type 2 diabetes. Diabetes.

[18] Zhang J, Liu Z, Li Z, Wang Y, Chen Y, Li X, Chen K, Shu N, Zhang Z (2016). Disrupted White Matter Network and Cognitive Decline in Type 2 Diabetes Patients. J Alzheimers Dis.

[19] van Bussel FC, Backes WH, van Veenendaal TM, Hofman PA, van Boxtel MP, Schram MT, Sep SJ, Dagnelie PC, Schaper N, Stehouwer CD, Wildberger JE, Jansen JF (2016). Functional Brain Networks Are Altered in Type 2 Diabetes and Prediabetes: Signs for Compensation of Cognitive Decrements? The Maastricht Study. Diabetes.

[20] Zalesky A, Fornito A, Bullmore ET (2010). Network-based statistic: identifying differences in brain networks. Neuroimage.

[21] Watts DJ, Strogatz SH (1998). Collective dynamics of ‘small-world’ networks. Nature.

[22] He Y, Chen ZJ, Evans AC (2007). Small-world anatomical networks in the human brain revealed by cortical thickness from MRI. Cereb Cortex.

[23] Hagmann P, Kurant M, Gigandet X, Thiran P, Wedeen VJ, Meuli R, Thiran JP (2007). Mapping human whole-brain structural networks with diffusion MRI. Plos One.

[24] Ferri R, Rundo F, Bruni O, Terzano MG, Stam CJ (2007). Small-world network organization of functional connectivity of EEG slow-wave activity during sleep. Clin Neurophysiol.

[25] Bassett DS, Meyer-Lindenberg A, Achard S, Duke T, Bullmore E (2006). Adaptive reconfiguration of fractal small-world human brain functional networks. Proc Natl Acad Sci USA.

[26] Salvador R, Suckling J, Coleman MR, Pickard JD, Menon D, Bullmore E (2005). Neurophysiological architecture of functional magnetic resonance images of human brain. Cereb Cortex.

[27] Bassett DS, Bullmore E (2006). Small-world brain networks. Neuroscientist.

[28] Sporns O, Zwi JD (2004). The small world of the cerebral cortex. Neuroinformatics.

[29] Stam CJ, Jones BF, Nolte G, Breakspear M, Scheltens P (2007). Small-world networks and functional connectivity in Alzheimer’s disease. Cereb Cortex.

[30] Zhang J, Wang J, Wu Q, Kuang W, Huang X, He Y, Gong Q (2011). Disrupted brain connectivity networks in drug-naive, first-episode major depressive disorder. Biol Psychiatry.

[31] Moran C, Phan TG, Chen J, Blizzard L, Beare R, Venn A, Munch G, Wood AG, Forbes J, Greenaway TM, Pearson S, Srikanth V (2013). Brain atrophy in type 2 diabetes: regional distribution and influence on cognition. Diabetes Care.

[32] Messier C (2005). Impact of impaired glucose tolerance and type 2 diabetes on cognitive aging. Neurobiol Aging.

[33] Manschot SM, Brands AM, van der Grond J, Kessels RP, Algra A, Kappelle LJ, Biessels GJ (2006). Brain magnetic resonance imaging correlates of impaired cognition in patients with type 2 diabetes. Diabetes.

[34] Xia W, Wang S, Rao H, Spaeth AM, Wang P, Yang Y, Huang R, Cai R, Sun H (2015). Disrupted resting-state attentional networks in T2DM patients. Sci Rep.

[35] Svacina S (2007). Olfaction and gustation in diabetes. Vnitr Lek.

[36] Naka A, Riedl M, Luger A, Hummel T, Mueller CA (2010). Clinical significance of smell and taste disorders in patients with diabetes mellitus. Eur Arch Otorhinolaryngol.

[37] Brady S, Lalli P, Midha N, Chan A, Garven A, Chan C, Toth C (2013). Presence of neuropathic pain may explain poor performances on olfactory testing in diabetes mellitus patients. Chem Senses.

[38] Chen YC, Xia W, Qian C, Ding J, Ju S, Teng GJ (2015). Thalamic resting-state functional connectivity: disruption in patients with type 2 diabetes. Metab Brain Dis.

[39] Cui Y, Jiao Y, Chen HJ, Ding J, Luo B, Peng CY, Ju SH, Teng GJ (2015). Aberrant functional connectivity of default-mode network in type 2 diabetes patients. Eur Radiol.

[40] Folstein MF, Folstein SE, McHugh PR (1975). “Mini-mental state”. A practical method for grading the cognitive state of patients for the clinician. J Psychiatr Res.

[41] Mayeda ER, Whitmer RA, Yaffe K (2015). Diabetes and cognition. Clin Geriatr Med.

[42] Jia J, Ning Y, Zhang J, Xu J, Wei W, Chen X (2014). The recommendation of diagnosis and treatment of cognitive impairment in Chinese elderly. Chin J Geriatr.

[43] Morris JC (1993). The Clinical Dementia Rating (CDR): current version and scoring rules. Neurology.

[44] Guo Q, Sun Y, Yu P, Hong Z, Lv C (2007). Norm of Auditory Verbal Learning Test in the normal aged in China community. Chinese Journal of Clinical Psychology.

[45] Suo X, Lei D, Li K, Chen F, Li F, Li L, Huang X, Lui S, Li L, Kemp GJ, Gong Q (2015). Disrupted brain network topology in pediatric posttraumatic stress disorder: A resting-state fMRI study. Hum Brain Mapp.

